# Systematic review protocol on COVID-19 vaccine hesitancy among lactating people

**DOI:** 10.3389/fgwh.2026.1828078

**Published:** 2026-09-16

**Authors:** Ephraim Senkyire, Gloria Senkyire, Ernestina Asiedua, Victor Tawose-Adebayo, Jude Kyeremeh Ameyaw, Rullmann Twi Owusu, Emmanuel Lamptey, Alex Asamoah, Magdalena Ohaja

**Affiliations:** 1 Ga West Municipal Hospital-Ghana Health Service, Department of Nursing and Midwifery, Amasaman-Accra, Ghana; 2 School of Nursing, University of British Columbia Okanagan, Kelowna, BC, Canada; 3 Department of Nursing and Midwifery, City St George's, University of London, London, United Kingdom; 4 Sunyani Technical University, Department of Accountancy, Faculty of Business and Management Studies, Sunyani, Ghana; 5 Department of Maternal and Child Health, University of Ghana, School of Nursing and Midwifery, Legon-Accra, Ghana; 6 Behavioural Training, Concept Care Disability, Sydney, NSW, Australia; 7 Elmina Polyclinic, Ghana Health Service, Department of Nursing and Midwifery, Cape Coast, Ghana; 8 University of Cape Coast, Department of Marketing and Supply Chain Management, School of Business, Cape Coast, Ghana; 9 University of Health and Allied Sciences, School of Basic and Biomedical Sciences, Ho, Ghana; 10 International Maritime Hospital, Department of Radiology, Tema-Accra, Ghana; 11 University of Galway, School of Nursing, Midwifery & Evidence Science, Galway, Irish Republic

**Keywords:** breastfeeding mothers, COVID-19, lactating people, systematic review, vaccine hesitancy

## Abstract

**Background:**

Lactating people experiencing SARS-CoV-2 infection encounter physical, emotional, and psychosocial challenges during the postpartum period compared to the general population. Despite the importance of COVID-19 vaccination globally, campaigns aimed at vaccinating at-risk populations, including pregnant and lactating people (PLP), have faced challenges, leading to a low uptake among PLP. The objectives of this study are to discover lactating people's perceptions toward COVID-19 vaccination uptake and to explore facilitators and barriers that inform their COVID-19 vaccination decision process.

**Methods:**

The search will be carried out using the MEDLINE (PubMed), CINAHL, Embase and Scopus databases. The search will utilise Medical Subject Headings (MeSH) terms and keywords to optimise results. The MeSH terms used will include ‘breastfeeding OR lactation’, ‘COVID-19 vaccine’, and ‘vaccination hesitancy’, along with various iterations of these keywords. Boolean operators ‘AND’ and ‘OR’ will be applied to expand the search scope. The search criteria will be limited to recent articles published between 2020 and 2026, in English, and with available abstracts. Two reviewers will independently screen titles/abstracts and full texts, while two reviewers will extract data. Study selection will adhere to the Preferred Reporting Items for Systematic Reviews and Meta-Analyses (PRISMA) protocol guidelines, and study quality will be evaluated using the Appraisal Tool for Cross-sectional Studies (AXIS), JBI qualitative checklist and MMAT.

**Result:**

Data will be analysed thematically following the approach outlined by Braun & Clarke (2006), presenting findings as a narrative summary.

**Conclusion:**

This review will help to design clear communication and targeted campaigns, building trust in vaccine safety and effectiveness.

**Ethics and dissemination:**

No formal ethical approval is necessary since this study does not involve the collection of primary data. The findings will be shared via a peer-reviewed journal article and presented at conferences.

**Clinical Trial registration number:** International Prospective Register for Systematic Reviews (PROSPERO) number CRD42024568930 (Version 1.5).

## 1 Introduction

The Coronavirus Disease 2019 (COVID-19) pandemic is a contagious illness triggered by the severe acute respiratory syndrome coronavirus 2 (SARS-CoV-2) (1). The World Health Organisation (WHO) in late 2019 discovered cases of novel coronavirus (nCoV) in China, and pronounced COVID-19 as a global public health concern in March 2020 (2, 3). As of July 2023, the global tally has surpassed 768 million confirmed cases and recorded 6.9 million deaths (4).

The global health crisis caused by COVID-19 witnessed the quickest vaccine distribution in history (1). The WHO now advises a simplified single-dose regimen for primary immunisation with most COVID-19 vaccines to enhance acceptance and uptake, offering sufficient protection as most people have been previously infected (1).

Data indicate that monovalent Omicron XBB vaccines offer slightly better protection compared to bivalent variant-containing and monovalent index virus vaccines (1). Nonetheless, in the absence of monovalent XBB vaccines, any WHO emergency-use-listed or prequalified vaccine remains beneficial, especially for high-risk groups (1). Therefore, it is crucial that everyone, everywhere, has access to COVID-19 vaccines (1).

Lactating people experiencing SARS-CoV-2 infection encounter physical, emotional, and psychosocial challenges during the postpartum period compared to the general population (5). Breastfeeding, recognised as a highly effective measure for preventing illness in mothers and children, involves the transfer of passive and active immunity through breast milk or vaccination (6, 7).

Despite the importance of COVID-19 vaccination globally, campaigns aimed at vaccinating at-risk populations, including pregnant and lactating people (PLP), have faced challenges, leading to a low uptake among PLP (2). Differing opinions (8) on the vaccination of lactating people against COVID-19 stem from concerns about viral transmission during lactation and the perceived risks and benefits of SARS-CoV-2 vaccines for lactating people (7).

World Health Organisation (WHO) (9) identified vaccine hesitancy as a risk to universal health nearly half a decade ago, defining it as the prolonged reluctance to accept safe vaccines despite available services (10). Factors contributing to vaccine hesitancy among PLPs include myths, costs, safety concerns, scepticism about healthcare providers and the health system, influence from community/religious leaders, and a lack of trust in government (10, 11).

The rapid advancement process of the COVID-19 vaccine and the exclusion of PLP from initial clinical trials led to vaccine hesitancy (7, 12–16). Vaccine hesitancy is especially prevalent in the context of COVID-19 as the speed of development and the use of novel vaccine technologies have increased concerns about possible adverse events and long-term outcomes (17).

According to recent research, COVID-19 vaccine hesitancy varies greatly based on knowledge, cultural beliefs, community COVID-19 transmission, and health and community services (18, 19). Vaccine hesitancy was found to be more common in young females and communities of colour (20), and vaccination data on over 20 million residents in the UK showed substantially lower vaccination rates in Black and financially deprived communities (21, 22).

Few studies have been published on actual vaccine uptake in PLP. Non-vaccination rates among PLPs can be as high as 48.4% (23), influenced by individual, interpersonal, healthcare system-level, and policy-level factors (24). In one of the only studies to examine vaccine acceptance in lactating people, only 38.6% were willing to receive the vaccine (25). The pooled overall COVID-19 vaccine hesitancy among lactating people in Bianchi et al.'s study was 53.5% compared to 49.2% for pregnant people (23). Moreover, the general rate of COVID-19 vaccine hesitancy from four cross-sectional studies was 52.5% - 85.4% among lactating people (2, 13, 26).

Numerous empirical studies (2, 13, 26–30) report COVID-19 vaccine hesitancy among PLPs, citing reasons such as anxiety, misconceptions, safety concerns, family influence, and infodemic.

An infodemic is characterised by an excessive amount of information, potentially containing inaccuracies or misinformation, spread across various digital and physical channels during a disease outbreak (31). For example, a qualitative study in Italy revealed that infodemic concerning safety, side effects, efficacy, and possible vaccine interference with other medical diseases were linked to vaccination hesitancy among participants (32). In parallel, similar concerns about hesitancy among women/people taking medication while lactating are well-documented (49, 50).

Contrary to hesitancy concerns, recent systematic studies find no serious vaccine-related effects on both mother and child (6). Current COVID-19 vaccines recommended for LPs utilise nonreplicating viral vectors, providing a safe option (2). Expert organisations, including WHO and the Academy of Breastfeeding Medicine, confirm vaccine safety and encourage post-vaccination breastfeeding, particularly as mRNA-related vaccines cannot be transmitted through breast milk (2, 26).

Furthermore, in a study aiming to ascertain the presence of vaccine-related mRNA in human milk following vaccination, it was found that none of the samples exhibited detectable levels of vaccine mRNA in any milk component (33). On the other hand, multiple studies have suggested a low risk, estimated between 2% to 3%, of vertical transmission and the presence of viral mRNA in breast milk among mothers infected with COVID-19 (44–48). However, some experts have theorised that immunoglobulin A induced by vaccination could pass through breast milk, potentially providing extra defence for infants against COVID-19 (34). Again, COVID-19 vaccination is considered safe during lactation and can enhance the transfer of virus-neutralising IgA and IgG antibodies through breast milk, potentially providing passive protection to breastfed infants (35). Similarly, evidence suggests that maternal vaccination or previous COVID-19 infection can confer protective antibodies to infants through breastfeeding (36). Despite this evidence, COVID-19 vaccine uptake among lactating people remains low. The necessity for a systematic review is to map partial peer-reviewed evidence, find research gaps, and inform future policy interventions. Therefore, this study aims to discover and explore lactating people's perceptions and experiences of the COVID-19 vaccine and answer the following questions: What are the perceptions of lactating people towards COVID-19 vaccination? What are the perceived barriers and facilitators of COVID-19 vaccine uptake among lactating people?

## 2 Methods and design

### 2.1 Population

The systematic review will focus on lactating people

### 2.2 Study design

This systematic review will consider cross-sectional, qualitative, and mixed-methods studies. The PICO framework (37) will guide the research questions and eligibility for this review. This review will adhere to the PRISMA-P guidelines (38). The PICO framework is defined as;

- Population (P): Lactating individuals, including studies involving pregnant and lactating populations where results for lactating participants are reported separately.
- Exposure/phenomenon of interest (I/E): COVID-19 vaccination and factors influencing vaccination decision-making, including perceptions, beliefs, confidence, safety concerns, information exposure, and other barriers and facilitators.
- Comparator (C): No comparator is required because the review focuses primarily on perceptions, experiences, barriers, facilitators, hesitancy, acceptance, intention, and uptake rather than comparative intervention effectiveness.
- Outcomes (O): COVID-19 vaccine hesitancy, acceptance/intention, refusal, uptake, and perceived barriers and facilitators.

### 2.3 Search strategy

The search will be conducted using the MEDLINE(PubMed), CINAHL, Embase and Scopus databases. A Librarian will review the search strategy to ensure it is complete and appropriate. Databases will facilitate Search optimisation using Medical Subject Headings (MeSH) terms and keywords. MeSH terms employed included ‘breastfeeding OR lactation ‘, ‘COVID-19 vaccine’, ‘vaccination hesitancy’, and keywords that were various iterations of these terms. The Boolean operators ‘AND’ and ‘OR’ will be incorporated alongside these MeSH and keyword terms to broaden the search scope. The search strategy will be taored to each database's syntax and subject headings. See Table 1.

**Table 1:** PUBMED search strategy.

**Concept 1: Breastfeeding/lactating**
**MeSH Terms:**
“Breast Feeding”[Mesh] OR “Lactation”[Mesh] OR “Postpartum Period”[Mesh]
**Keywords:**
“Breast Fed*” OR “Exclusive Breast Feeding” OR “Breastfed” OR “Breastfeeding” OR “lactating” OR “lactat*” OR “lactating” OR “lactation” OR “Exclusive Breastfeeding” OR “postpartum” OR “post-partum” OR “postnatal” OR “nursing mother*” OR “breastfeeding mother*” OR “lactating women”
**Concept 2: COVID-19**
**MeSH Terms:**
“COVID-19”[Mesh] OR “SARS-CoV-2”[Mesh] OR “COVID-19 Vaccines”[Mesh]
**Keywords:**
“2019 Novel Coronavirus Vaccine*” OR “2019-nCoV Vaccine*” OR “COVID 19 Vaccine*” OR “COVID-19 Vaccine*” OR “COVID-19 Virus Vaccine*” OR “COVID19 Vaccine*” OR “COVID19 Virus Vaccine*” OR “Coronavirus Disease 2019 Vaccine*” OR “Coronavirus Disease 2019 Virus Vaccine*” OR “Coronavirus Disease-19 Vaccine*” OR “SARS Coronavirus 2 Vaccine*” OR “SARS-CoV-2 Vaccine*” OR “SARS2 Vaccine*”
**Concept 3: Vaccine hesitancy**
**MeSH Terms:**
“Vaccination Hesitancy”[Mesh] OR “Vaccination”[Mesh] OR “Immunisation”[Mesh]
**Keywords:**
“Vaccination Delay” OR “Vaccine Delay” OR “Vaccine Hesitancy” OR Vaccine acceptance OR Vaccine uptake OR Vaccine rejection OR vaccine hesitan* OR vaccine reluctan* OR vaccine refusal OR vaccine accept* OR vaccine attitude* OR vaccine confidence OR vaccine uptake OR vaccine intent* OR vaccination willingness OR immunization hesitan* OR vaccination barrier* OR vaccine perception*
**Concept 1 AND Concept 2 AND Concept 3**
**Limiters: English language, 2020-2026**

### 2.4 Inclusion and exclusion criteria

The inclusion criteria will consist of studies involving lactating people, cross-sectional, qualitative and mixed-methods studies that report on COVID-19 vaccine perception, barriers, and facilitators among lactating people, studies published between 2020 and 2026in English, and peer-reviewed articles. This time frame indicates a substantial surge in the recording of COVID-19 vaccine hesitancy. Studies that include both pregnant and lactating people but report separate results for lactating people will also be considered. The exclusion criteria will include studies with only an available abstract, editorials, preprints, books, conference papers, theses, and case reports.

### 2.5 Information sources

These will include searches on MEDLINE(PubMed), Embase, Scopus, and CINAHL (EBSCOhost). Titles and abstracts will be screened for relevance, with search parameters such as date ranges, keywords, and advanced search tools used to minimise the omission of relevant studies. Full texts of eligible articles will then be retrieved, and duplicate records will be removed before screening. To enhance comprehensiveness, the reference lists of included studies, as well as related systematic reviews, will be examined to identify additional relevant sources.

### 2.6 Study selection

The study selection process will be reported using the four stages of the PRISMA flowchart and three reviewers (E.S, G.S and M.O) will independently assess the articles. Before commencing formal screening, all reviewers involved in study selection will undertake a calibration exercise using a small sample of retrieved records. The reviewers will independently assess the same titles and abstracts against the predefined eligibility criteria and subsequently compare their decisions. Any differences in interpretation will be discussed and resolved by consensus to ensure a common understanding of the inclusion and exclusion criteria. The eligibility criteria and screening procedures will be refined where necessary before formal screening begins.

Following calibration, titles and abstracts will be screened independently by three reviewers against the predefined eligibility criteria. Records considered potentially eligible by either reviewer will proceed to full-text assessment. Full texts will then be independently assessed by the reviewers to determine final eligibility. Reasons for exclusion at the full-text stage will be documented and reported in the PRISMA flow diagram. Any disagreement between reviewers at either the title/abstract or full-text stage will initially be discussed between the reviewers to reach consensus. Where consensus cannot be achieved through discussion, a fourth reviewer will independently assess the record and participate in the resolution process. The final decision will be based on discussion and agreement among the reviewers, with the decision and reason documented to maintain an auditable selection process.

### 2.7 Quality assessment

Studies will be assessed independently by three reviewers (E.S, G.S and M.O) using the Appraisal Tool for Cross-sectional Studies (AXIS) (39), the JBI checklist for qualitative studies (40), and Mixed Method Appraisal Tool (MMAT) for mixed-methods studies (41). Before formal quality assessment, reviewers will independently appraise a small number of eligible studies to calibrate their interpretation and application of the relevant appraisal criteria. Disagreements will be discussed and resolved by consensus. If consensus cannot be reached, a fourth reviewer will be consulted. The appraisal process and any disagreements will be documented.

### 2.8 Operational definitions

- Vaccine hesitancy: delay in acceptance or reluctance/refusal despite availability of vaccination.
- Vaccine acceptance/intention: stated willingness or intention to receive a COVID-19 vaccine.
- Vaccine refusal: an explicit decision not to receive vaccination.
- Vaccine uptake: actual receipt of vaccination.
- Vaccine confidence: trust in vaccine safety, effectiveness, healthcare providers, and vaccination systems.
- Barriers/facilitators: individual, interpersonal, community, healthcare-system, service-delivery, and policy factors that hinder or support vaccination.

### 2.8 Data extraction

Detailed characteristics of eligible studies will be manually extracted by two authors (E.S and G.S) and categorised using a predefined template developed by the researcher (E.S) and validated by an independent researcher (E.A). Two reviewers will independently pilot the form using a small sample of eligible studies to determine whether the variables adequately capture the characteristics, outcomes, contextual factors, and methodological information required for the review. The form will be revised where necessary following discussion among the review team. The characteristics extracted from the studies include authors, year, country, study aim, design, sample, data collection, findings and limitations related to COVID-19 vaccine hesitancy among lactating individuals. See Table 2. Discrepancies in extracted data will be compared and discussed between the reviewers. Where differences cannot be resolved through discussion, a fourth reviewer will be consulted. Changes made to the extraction form following pilot testing will be documented to ensure transparency.

**Table 2:** Data extraction.

Author, year, country	Aim	Design/ Methodology	Data collection	Sample size	Key findings	Limitations
						

### 2.9 Outcomes

The primary outcome assessed will be:

1. COVID-19 vaccine hesitancy;
2. COVID-19 vaccination acceptance/intention;
3. COVID-19 vaccination uptake/refusal, where reported.

The secondary outcomes will include:

1. Perceived barriers to vaccination;
2. Perceived facilitators of vaccination;
3. Vaccine confidence and trust;
4. Perceived vaccine safety and adverse effects;
5. Perceived vaccine effectiveness;
6. Knowledge and beliefs concerning COVID-19 vaccination;
7. Sources and quality of vaccination information;
8. Healthcare-provider recommendations;
9. Access and service-delivery barriers; and
10. Individual, interpersonal, community, and health-system factors influencing vaccination decisions.

### 2.10 Missing or unclear information

Where important information required for eligibility assessment, quality appraisal, or data extraction is missing or unclear, the reviewers will first examine the full publication and any supplementary materials available from the original study. Where feasible, the corresponding author or study authors will be contacted to request clarification or additional information. A reasonable period will be allowed for response.

If clarification cannot be obtained, the information will be recorded as not reported, unclear, or not available, as appropriate. Reviewers will not infer or impute information that cannot be reliably established from the available evidence. The number and nature of studies with important missing information, attempts to contact authors, and responses received will be documented where relevant. The potential implications of missing information for the interpretation of the findings will be considered during synthesis.

### 2.10 Analysis

The final review will feature a narrative summary of the quantitative findings from the selected studies. Quantitative findings will initially be examined for comparability in terms of population, exposure, outcome definitions, measurement instruments, study design, and contextual characteristics. A meta-analysis will be considered if a sufficiently homogeneous group of studies reports comparable outcomes using sufficiently similar definitions and measures. Thus, where quantitative pooling is inappropriate because of substantial clinical, methodological, or statistical heterogeneity, findings will be synthesised narratively. For qualitative findings, we will use a systematic thematic synthesis approach (42) Coding will be undertaken by designated reviewers and will initially allow themes to emerge inductively from the evidence. The synthesis will subsequently consider patterns across individual, interpersonal, community, healthcare-system, and service-delivery factors. Quantitative and qualitative findings will be integrated at the interpretive level while maintaining the distinction between numerical estimates and contextual or experiential evidence.

The certainty of evidence will be assessed where appropriate to the nature of the available evidence and the review outcomes. For quantitative outcomes, certainty will be considered in relation to methodological limitations, consistency of findings, directness, precision, and other relevant factors. For qualitative findings, confidence in the synthesised findings will be considered using an approach appropriate to qualitative evidence. Because the review is expected to include diverse study designs and outcome measures, certainty or confidence assessments will be applied according to the type of evidence and outcome rather than assuming that a single approach is appropriate for all included studies.

The certainty assessment will be used to distinguish findings supported by relatively consistent and credible evidence from findings where substantial methodological limitations, inconsistency, indirectness, imprecision, or insufficient information warrant greater caution. This assessment will inform the interpretation of the review findings and the strength of conclusions and recommendations. The synthesis approach will reflect the methodological and clinical diversity of the included evidence. Where sufficient homogeneity exists, quantitative findings may be statistically synthesised. Where substantial heterogeneity precludes meaningful pooling, findings will be presented using a structured narrative synthesis.

## 3 Conclusion

This review will contribute to the evidence around vaccine hesitancy among lactating people and the design of context-specific messages in promoting vaccine uptake among lactating people.

## 4 Implications

There is a notable lack of research on the perceptions and experiences of lactating individuals regarding COVID-19 vaccine hesitancy globally. Addressing the determinants of vaccine hesitancy is essential to improving vaccine acceptance and uptake in this population. Beyond developing context-specific strategies and messaging, the findings could inform interventions that strengthen the role of trusted healthcare professionals in providing accurate, individualised vaccination information and recommendations. For lactating individuals, opportunities to discuss and receive COVID-19 vaccination should extend beyond obstetric services to include primary care, paediatric services, pharmacies, and other accessible community healthcare settings. Integrating vaccination into routine maternal, postpartum, and family healthcare may help reduce missed opportunities and address practical barriers to uptake. Evidence from recent vaccination research also suggests that pharmacist-led interventions can improve vaccination coverage among vulnerable and underserved populations (43). Accordingly, future interventions should combine effective communication with accessible vaccination services, trusted healthcare-provider engagement, and convenient delivery pathways to support informed COVID-19 vaccination decisions among lactating individuals.

## 5 Strengths and limitations

This review will be the first systematic review examining COVID-19 vaccine perceptions, barriers, and facilitators among lactating people. The PRISMA guidelines will guide the review process to ensure transparency and rigorous reporting, and all included studies will be appraised using an appropriate quality assessment tool.

Several methodological limitations are anticipated. Selection bias may affect the generalisability of the findings. The review will also be restricted to English-language publications, which may result in the exclusion of relevant non-Anglophone and non-indexed local literature. In addition, the omission of grey literature searches may lead to the exclusion of pertinent reports, policy documents, or local evidence. There is also a potential risk of publication bias, as studies documenting the phenomenon may be more likely to be published than those reporting lower prevalence. Restricting the evidence base primarily to peer-reviewed literature may introduce publication bias, as studies reporting statistically significant, favourable, or otherwise notable findings may be more likely to reach publication than studies reporting null or less prominent findings. This may result in an overrepresentation of particular perceptions, barriers, or facilitators of COVID-19 vaccination among lactating individuals and consequently affect the balance of the evidence synthesised.

Database-related retrieval bias may affect the comprehensiveness of the evidence identified. Although the search strategy uses major biomedical and nursing databases and reference-list screening is planned, studies indexed exclusively in other databases or published in less commonly indexed sources may remain unidentified. Consequently, the evidence captured may not fully represent the global range of experiences and determinants influencing COVID-19 vaccination among lactating individuals.

Substantial conceptual and measurement heterogeneity is anticipated across the included studies. Vaccine hesitancy, acceptance, intention, refusal, and actual vaccine uptake are related but distinct constructs, and studies may define and measure them differently. For example, stated willingness to receive a vaccine may not necessarily translate into actual vaccination, particularly when access or healthcare-system barriers are present. Such variation may make direct comparison of prevalence estimates and determinants difficult and may limit the extent to which findings can be meaningfully combined.

Clinical, contextual, and methodological heterogeneity across countries, healthcare systems, vaccine types, and study designs may further influence the findings. Differences in national vaccination policies, availability of vaccines, public-health messaging, healthcare access, cultural contexts, and timing of vaccination campaigns may shape vaccine perceptions and uptake independently of individual hesitancy. Similarly, differences between quantitative observational studies and qualitative studies in sampling, data collection, outcome measurement, and analytical approaches may produce findings that are complementary but not directly comparable. Therefore, apparent differences between studies should be interpreted cautiously and within their respective contextual and methodological settings.

The inclusion and synthesis of both qualitative and quantitative evidence may present challenges in integrating different forms of knowledge. Quantitative studies may estimate the prevalence or strength of associations, whereas qualitative studies may provide contextual explanations of experiences, beliefs, and decision-making processes. These forms of evidence answer related but different questions and cannot always be directly combined. The synthesis will therefore emphasise patterns and convergence across evidence types while avoiding inappropriate equivalence between numerical estimates and qualitative themes.

Because the evidence is predominantly observational, the review will be unable to establish causal relationships between identified factors and COVID-19 vaccination outcomes. Cross-sectional studies can identify associations, patterns, and contextual relationships, but they cannot establish that a particular factor directly caused vaccine hesitancy or non-uptake. For example, an association between safety concerns and vaccine non-uptake does not necessarily demonstrate that safety concerns independently caused the decision not to vaccinate, as other individual, interpersonal, healthcare-system, or contextual factors may operate simultaneously. The findings will therefore be interpreted as evidence of associations and contributing factors rather than causal determinants.

Finally, the completeness and availability of information on postpartum and healthcare-system characteristics may be inconsistent across studies. Variables such as time since delivery, parity, previous COVID-19 infection or vaccination, maternal education, healthcare-worker status, provider recommendation, accessibility of vaccination services, and integration of vaccination into routine healthcare may not be consistently reported. Missing or poorly reported information may limit the ability to examine how postpartum circumstances and health-system factors interact with individual perceptions and vaccination decisions. This may also constrain the development of context-specific recommendations and reduce the extent to which findings can be translated across different healthcare settings.
